# Adenoviruses found in bats of southwestern Texas

**DOI:** 10.1007/s00705-025-06499-9

**Published:** 2025-12-18

**Authors:** Jadance L. Black, Alexandra Moya, Matt J. Van Sant, Dana N. Lee

**Affiliations:** Department of Agriculture, Biology, & Health Sciences, Cameron University, 2800 W. Gore Blvd, 73505 Lawton, OK USA

## Abstract

Few studies have examined adenoviruses (AdVs) in bats from the U.S.; therefore, we targeted a fragment of the adenoviral DNA polymerase gene from 94 fecal samples from nine bat species in Texas. Three samples from *Antrozous pallidus* had identical sequences, but these differed from those found in one *Myotis yumanensis* and one *Tadarida brasiliensis* sample. Phylogenetic analysis showed that these DNA sequences were most similar to those of a previously described AdV found in *Myotis velifer* in Oklahoma. Each of these sequences differed by more than > 12% from those of any currently classified AdVs and by more than 10% from that of a currently unclassified AdV isolate from *Myotis myotis.*

**Supplementary Information:**

The online version contains supplementary material available at 10.1007/s00705-025-06499-9.

Bats make up the second-most diverse mammalian order, with over 1,300 known species belonging to 21 different families [1, 2]. There are significant differences in size, morphology, ecological niches, diet, and social interactions between the two suborders [1, 2]. Although many studies have been conducted on various viruses that infect bats, relatively few have focused on DNA viruses, such as adenoviruses (AdVs). AdVs are non-enveloped, double-stranded DNA viruses that infect members of nearly all of the major vertebrate classes [3], with bats serving as an important reservoir of diverse adenovirus lineages [4–6]. Of the six genera in the family *Adenoviridae*, members of the genus *Mastadenovirus* are found in mammals, including bats. Members of 16 mastadenovirus species recognized by the International Committee on Taxonomy of Viruses (ICTV) have been found in bats: *Mastadenovirus musauriti, Mastadenovirus pipistrelli, Mastadenovirus rhinolopidae, Mastadenovirus miniopteridae, Mastadenovirus humile, Mastadenovirus pteropodidae, Mastadenovirus magnauris, Mastadenovirus eidoli, Mastadenovirus aegyptiaci, Mastadenovirus asiensse, Mastadenovirus cardiodermatis, Mastadenovirus chalinolobi, Mastadenovirus desmodi, Mastadenovirus vespertilionis, Mastadenovirus fructus*, and *Mastadenovirus canidae* [3, 5–19].

Bats are reservoirs for many AdVs that can potentially be zoonotic [20–22], some of which can cause serious diseases in humans despite causing no clinical signs of infection in bats [21]. There are many hypotheses as to why bats can carry viruses without showing signs of disease. One hypothesis is that bats coevolved with their viruses to shape their unique immunological responses [2]. While bats have many mechanisms to prevent disease, humans are not as protected in some cases. It has been estimated that about 60–75% of emerging infectious diseases of humans originate from animals, including mammals [23]. Bats have been implicated as reservoirs or ancestral hosts for several high-impact zoonoses, including filoviruses such as Ebola virus and SARS-like coronaviruses; MERS-CoV circulates in camels but is likely to have originated from bats [23–25]. Chen et al. [26] demonstrated cross-species transmission of titi monkey adenovirus (*Mastadenovirus simuli*), a novel virus that is capable of infecting both monkeys and humans. While the potential for zoonosis is alarming, it is thought that the rate of transmission of AdVs from bats to humans is actually low [27].

Most research investigating the diversity of AdVs in bats has taken place in the Eastern Hemisphere [7, 15, 18, 28, 29], and only a few studies from North America have been published [14, 30–32]. In this study, we sampled nine different bat species in Big Bend National Park, Texas: *Aeorestes cinereus*, *Antrozous pallidus*, *Corynorhinus townsendii*, *Eptesicus fuscus*, *Mormoops megalophylla*, *Parastrellus hesperus, Myotis velifer*, *Myotis yumanensis*, and *Tadarida brasiliensis*.

We captured bats using mist nets and harp traps on nine nights from May to July 2023. All of the bats were handled following the guidelines of Sikes et al. [33], and white nose syndrome decontamination protocols were followed during this process [34]. Captured bats were placed individually in breathable cloth bags for < 30 minutes, and fecal samples were collected from the bags using sterile forceps. The samples from 94 bats were placed into RNAlater upon collection and were stored at −20ºC until DNA extraction.

DNA was extracted using a QIAamp Stool Mini Kit, following the manufacturer’s protocol, except a longer incubation time at 70°C was used. After extraction, nested PCR targeting a fragment from the adenoviral DNA polymerase gene was performed on each of the samples according to Li et al. [5], using the primers pol-F (5’ CAGCCKCKGTTRTGYAGGGT 3’) and pol-R (5’ GCHACCATYAGCTCCAACTC 3’). The cycling profile consisted of 94ºC for 5 min; 30 cycles of 94ºC for 30 s, 50ºC for 30 s, and 72ºC for 30 s, and a final extension of 72ºC for 10 min. The second round of PCR was performed using 1 µl of the first PCR product as a template, the primers pol-nf (5’ GGGCTCRTTRGTCCAGCA 3’) and pol-nr (5’ TAYGACATCTGYGGCATGTA 3’), and the same cycle profile used for the first round of PCR. Both positive (DNA from human adenovirus type 1D, American Type Culture Collection) and negative controls (no DNA present) were used for each round of PCR. After identifying the positive PCR products via gel electrophoresis, the samples were purified using ExoSAP-IT Express PCR Product Cleanup following the manufacturer's protocol.

Next, Sanger sequencing of the positive samples for AdV species identification was performed by the Texas A&M Corpus Christi Genomics Lab, and the sequences were aligned and manually edited in Geneious v. 2025.2 [35]. Sequences from isolates representing other adenovirus species, obtained from bats (*M. musauriti, M. pipistrelli, M. rhinolopidae, M. miniopteridae, M. humile, M. pteropodidae, M. magnauris, M. eidoli, M. aegyptiaci, M. asiensse, M. cardiodermatis, M. chalinolobi, M. desmodi, M. vespertilionis, M. fructus, M. canidae*, and the proposed but not yet approved species "*Mastadenovirus portugalense*", "*Mastadenovirus himalaiense*", "*Mastadenovirus kuhlii*", "*Mastadenovirus noctulae*", "*Mastadenovirus ferrumequini*", "*Mastadenovirus arundinis*"), turkeys (*Siadenovirus gallopavotertii*), canines (*Mastadenovirus canidae*), bovines (*Mastadenovirus bostertium*), and humans (*Mastadenovirus caesari* [HAdV-2], *Mastadenovirus blackbeardi* [HAdV-3], *Mastadenovirus adami* [HAdV-12], and *Mastadenovirus dominans* [HAdV-9]) in GenBank were added to the final alignment. MEGA 12 [36] was used to perform phylogenetic analysis by the maximum-likelihood method, with 1000 bootstrap replicates. We followed the current convention for AdV species names approved by the ICTV [3] as well as for names of proposed species that have not yet been approved [37–40].

Out of 94 fecal samples (two from *A. cinereus*, 36 from *A. pallidus*, three from *C. townsendii*, three from *E. fuscus*, four from *M. megalophylla*, two from *M. velifer*, five from *M. yumanensis*, nine from *P. hesperus*, and 30 from *T. brasiliensis*), six were positive for an AdV, but we were able to obtain sequence data from only five of them. The positive samples that we sequenced were isolated from *A. pallidus* (fecal samples 28, 31, and 72), *T. brasiliensis* (fecal sample 61), and *M. yumanensis* (fecal sample 70). Phylogenetic analysis using the Le and Gascuel (LG) + G + I model showed that the five viruses belonged to the same clade as other mastadenoviruses (Fig. 1). The sequences from samples 28, 31, and 72 were identical to each other but differed by 12.5% and 9.1% at the amino acid level compared to those from samples 61 and 70, respectively (Table 1). The sequences from samples 61 and 70 differed by 4.5% from each other. Interestingly, the AdV DNA sequences found in the bats from Texas were most similar to those found in *M. velifer* in Oklahoma [30], differing in their predicted amino acid sequences by 5.7–10.2%. The viruses from the bats we collected in Texas and the bats collected in Oklahoma [30] formed a monophyletic group that was separate from a virus found in *Myotis myotis* in Portugal [39] (Fig. 1) for which the species name "*Mastadenovirus portugalense*" has been proposed and is awaiting approval by the ICTV [3, 37]. The partial predicted amino acid sequences of the viruses in the Texas samples differ by at least 10.2% from the corresponding sequence from the Portuguese isolate and at least 12.5% from those of previously identified adenoviruses (Table 1).

**Table 1:** Percent amino acid sequence difference in the 264-bp-long PCR products obtained from the DNA polymerase gene of putative adenoviruses in bat samples

Mastadenovirus species or sample	Bat species	GenBank ID	Fecal 28, 31, 72	Fecal 61	Fecal 70
*Mastadenovirus musauriti*	*Myotis ricketti*	GU226970 [5]	21.6	23.9	19.3
*Mastadenovirus pipistrelli*	*Pipistrellus pipistrellus*	JN252129 [11]	23.9	26.1	21.6
*Mastadenovirus rhinolopidae*	*Rhinolophus sinicus*	KT698853 [12]	27.3	28.4	23.9
*Mastadenovirus miniopteridae*	*Miniopterus schreibersii*	KT698856 [13]	31.8	36.4	31.8
*Mastadenovirus humile*	*Miniopterus schreibersii*	KT698852 [13]	29.5	30.7	26.1
*Mastadenovirus pteropodidae*	*Rousettus leschenaultia*	KX961095 [13]	35.2	36.4	31.8
*Mastadenovirus magnauris*	*Corynorhinus rafinesquii*	KX871230 [14]	21.6	25.0	20.5
*Mastadenovirus eidoli*	*Eidolon helvum*	AP018374 [9]	34.1	35.2	30.7
*Mastadenovirus aegyptiaci*	*Rousettus aegyptiacus*	MG551742 [10]	30.7	35.2	30.7
*Mastadenovirus asiensse*	*Vespertilio sinensis*	LC385827 [8]	21.6	25.0	20.5
*Mastadenovirus cardiodermatis*	*Cardioderma cor*	PP711818 [15]	25.0	26.1	21.6
*Mastadenovirus chalinolobi*	*Chalinolobus gouldii*	MK472072 [16]	18.2	18.2	13.6
*Mastadenovirus desmodi*	*Desmodus rotundus*	BK066905 [17]	20.5	20.5	15.9
*Mastadenovirus vespertilionis*	Unspecified bat	BK066631 [17]	15.9	18.2	13.6
*Mastadenovirus fructus*	*Rousettus leschenaultii*	OR998962 [18]	17.0	17.0	12.5
*Mastadenovirus canidae*	*Eptesicus serotinus*	KM043107 [19]	21.6	22.7	20.5
"*Mastadenovirus ferrumequini*"*	*Rhinolophus ferrumequinum*	PP410069 [38]	23.9	26.1	21.6
"*Mastadenovirus portugalense*"*	*Myotis myotis*	PV383552 [39]	14.8	14.8	10.2
"*Mastadenovirus himalaiense*"*	*Myotis siligorensis*	OR998961 [18]	17.0	17.0	12.5
"*Mastadenovirus noctulae*"*	*Nyctalus noctula*	PP297886 [40]	14.8	15.9	11.4
"*Mastadenovirus kuhlii*"*	*Pipistrellus kuhlii*	PP410068 [3]	18.2	19.3	14.8
"*Mastadenovirus arundinis*"*	*Tylonycteris robustula*	OR998870 [18]	31.8	33.0	28.4
Guano sample 2***	*Myotis velifer*	MN240005 [30]	29.5	31.8	28.4
Guano sample 61***	*Myotis velifer*	MN240006 [30]	27.3	28.4	26.1
Guano sample 21***	*Myotis velifer*	MN240007 [30]	10.2	10.2	5.7
Fecal sample 28, 31, 72**	*Antrozous pallidus*	PQ083675		12.5	9.1
Fecal 61**	*Tadarida brasiliensis*	PQ083676	12.5		4.5
Fecal 70**	*Myotis yumanensis*	PQ083677	9.1	4.5	

**Fig. 1:**
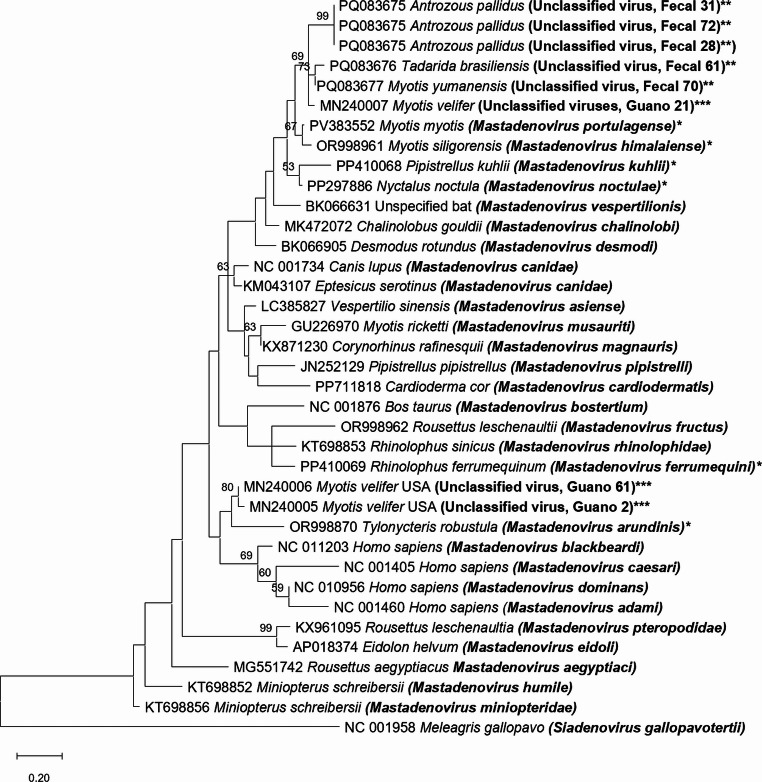
Phylogenetic analysis based on an 88-amino-acid-long fragment of the viral DNA polymerase. The tree was constructed by the maximum-likelihood method and rooted using turkey adenovirus type 3, a member of the genus *Siadenovirus*, as an outgroup. The tree was constructed using the Le and Gascuel + G + I model and 1000 bootstrap replicates. Bootstrap values > 50 are shown. Reference sequences representing different adenovirus species were retrieved from the GenBank database. Branches are labelled with GenBank accession numbers and host species name, with virus species name in parentheses. *, proposed but yet not approved adenovirus species; **, putative adenoviruses detected in the present work; ***, putative bat adenoviruses detected in the USA earlier by Lee and Angiel [30]

The DNA sequences obtained from the viruses found in *A. pallidus*, *T. brasiliensis*, and *M. yumanensis* in this study have been deposited in the GenBank database under the accession numbers PQ083675-PQ083677. While these sequences are very short (264 base pairs) and the results therefore must be considered preliminary, it is likely that they represent a novel adenovirus that is circulating in bats in the Oklahoma/Texas region of North America. Whole-genome sequencing of AdVs from positive bats from this region is needed to confirm this.

It is important to keep searching for viruses found in different bat species because of their potential to be transmitted to other hosts. For example, fecal samples 61 and 70 from our study were from bats of different families but appeared to contain the same virus. Other studies have found evidence that not all Advs are strictly species-specific, and this is particularly true of bat AdVs [5, 41]. In fact, a bat mastadenovirus has been shown to replicate in canine, simian, and human cell lines [5], and a previous genomic analysis revealed that a bat adenovirus shared close sequence and structural similarity with a canine adenovirus, both of which are now classified as members of the species *Mastadenovirus canidae* [3], suggesting possible evolutionary links among mammalian adenoviruses [6]. Li et al. [5] have shown that some bat AdVs have a similar GC content and show amino acid sequence similarity in their structural proteins to human AdVs. Human adenovirus type 5, which belongs to the species *M. caesari*, has been developed and tested as a vector for gene therapy and vaccine delivery in China and other countries, and it is possible that bat adenovirus could also be useful tools in human medicine. This study highlights the importance of maintaining surveillance programs for AdVs in bats and suggests that sequencing the entire genomes of these viruses is warranted in order to expand our knowledge of their genetic diversity and evolution.

## Electronic Supplementary Material

Below is the link to the electronic supplementary material

Supplementary Material 1 (DOCX 16.3 KB)
