# Supplementary material for: Adenoviruses found in bats of southwestern Texas
Source: Arch Virol. 2025 Dec 18;171(1):22. doi: 10.1007/s00705-025-06499-9 (PMC12975823; doi:10.1007/s00705-025-06499-9)
Supplement: Supplementary file 1 — Supplementary Material 1 (DOCX 16.3 KB) [file 705_2025_6499_MOESM1_ESM.docx]

GenBank flat file:
LOCUS       PQ083675                 258 bp    DNA     linear   VRL 26-JUL-2024
DEFINITION  Bat mastadenovirus isolate Guano 28 DNA polymerase gene, partial
           cds.
ACCESSION   PQ083675
VERSION     PQ083675
KEYWORDS    .
SOURCE      Bat mastadenovirus
 ORGANISM  Bat mastadenovirus
           Viruses; Varidnaviria; Bamfordvirae; Preplasmiviricota;
           Tectiliviricetes; Rowavirales; Adenoviridae; Mastadenovirus.
REFERENCE   1  (bases 1 to 258)
 AUTHORS   Lee,D.N. and Black,J.L.
 TITLE     Adenoviruses Found in Bats in West Texas
 JOURNAL   Unpublished
REFERENCE   2  (bases 1 to 258)
 AUTHORS   Lee,D.N. and Black,J.L.
 TITLE     Direct Submission
 JOURNAL   Submitted (25-JUL-2024) Agriculture, Biology, and Health Sciences,
           Cameron University, 2800 W Gore Blvd, Lawton, OK 73505, USA
COMMENT     ##Assembly-Data-START##
           Sequencing Technology :: Sanger dideoxy sequencing
           ##Assembly-Data-END##
FEATURES             Location/Qualifiers
    source          1..258
                    /organism="Bat mastadenovirus"
                    /mol_type="genomic DNA"
                    /isolate="Guano 28"
                    /isolation_source="feces"
                    /host="Antrozous pallidus"
                    /db_xref="taxon:740971"
                    /geo_loc_name="USA: Texas"
    CDS             complement(<1..>258)
                    /codon_start=1
                    /product="DNA polymerase"
                    /protein_id="XDG10646"
                    /translation="DICGMYASALTHPFPAGRPLNPLDRALAVGRYESMMKTNKTLCY
                    FDRALLPAIFTIDADPPDEHLLDVLPPFCSRKGGRLCWTNEP"
ORIGIN      
       1 gggctcgttg gtccagcaca gacgaccgcc cttcctggag cagaacggcg gcagcacatc
      61 gaggagatgc tcgtccgggg ggtcggcgtc tatcgtgaag atggccggga gtagtgcgcg
     121 atcgaaataa cataacgttt tgtttgtttt catcatgctt tcgtaccgtc ccaccgcgag
     181 cgctcggtcg agagggttga ggggcctgcc cgctgggaac gggtgggtga gggctgatgc
     241 atacatgccr cagatgtc
//

LOCUS       PQ083676                 201 bp    DNA     linear   VRL 26-JUL-2024
DEFINITION  Bat mastadenovirus isolate Guano 61 DNA polymerase gene, partial
           cds.
ACCESSION   PQ083676
VERSION     PQ083676
KEYWORDS    .
SOURCE      Bat mastadenovirus
 ORGANISM  Bat mastadenovirus
           Viruses; Varidnaviria; Bamfordvirae; Preplasmiviricota;
           Tectiliviricetes; Rowavirales; Adenoviridae; Mastadenovirus.
REFERENCE   1  (bases 1 to 201)
 AUTHORS   Lee,D.N. and Black,J.L.
 TITLE     Adenoviruses Found in Bats in West Texas
 JOURNAL   Unpublished
REFERENCE   2  (bases 1 to 201)
 AUTHORS   Lee,D.N. and Black,J.L.
 TITLE     Direct Submission
 JOURNAL   Submitted (25-JUL-2024) Agriculture, Biology, and Health Sciences,
           Cameron University, 2800 W Gore Blvd, Lawton, OK 73505, USA
COMMENT     ##Assembly-Data-START##
           Sequencing Technology :: Sanger dideoxy sequencing
           ##Assembly-Data-END##
FEATURES             Location/Qualifiers
    source          1..201
                    /organism="Bat mastadenovirus"
                    /mol_type="genomic DNA"
                    /isolate="Guano 61"
                    /isolation_source="feces"
                    /host="Tadarida brasiliensis"
                    /db_xref="taxon:740971"
                    /geo_loc_name="USA: Texas"
    CDS             complement(<1..>201)
                    /codon_start=1
                    /product="DNA polymerase"
                    /protein_id="XDG10647"
                    /translation="DXSGMYGSAAPHPFPAGRPLNPFDRALAVHRYEAKMKTCKTMDY
                    FDSALLPAIFTIDADPPDEHLLD"
ORIGIN      
       1 gtccagcaag tgttcgtccg gagggtcggc gtcgatggtg aagatggccg gcagaagagc
      61 ggagtcaaag tagtccatgg ttttgcaagt cttcatcttg gcttcgtacc tgtgcacggc
     121 gagcgctcgg tcgaacgggt tgaggggtcg ccccgcgggg aagggatgag gggcagccga
     181 accgtacatg ccgctantgt c
//
LOCUS       PQ083677                 201 bp    DNA     linear   VRL 26-JUL-2024
DEFINITION  Bat mastadenovirus isolate Guano 70 DNA polymerase gene, partial
           cds.
ACCESSION   PQ083677
VERSION     PQ083677
KEYWORDS    .
SOURCE      Bat mastadenovirus
 ORGANISM  Bat mastadenovirus
           Viruses; Varidnaviria; Bamfordvirae; Preplasmiviricota;
           Tectiliviricetes; Rowavirales; Adenoviridae; Mastadenovirus.
REFERENCE   1  (bases 1 to 201)
 AUTHORS   Lee,D.N. and Black,J.L.
 TITLE     Adenoviruses Found in Bats in West Texas
 JOURNAL   Unpublished
REFERENCE   2  (bases 1 to 201)
 AUTHORS   Lee,D.N. and Black,J.L.
 TITLE     Direct Submission
 JOURNAL   Submitted (25-JUL-2024) Agriculture, Biology, and Health Sciences,
           Cameron University, 2800 W Gore Blvd, Lawton, OK 73505, USA
COMMENT     ##Assembly-Data-START##
           Sequencing Technology :: Sanger dideoxy sequencing
           ##Assembly-Data-END##
FEATURES             Location/Qualifiers
    source          1..201
                    /organism="Bat mastadenovirus"
                    /mol_type="genomic DNA"
                    /isolate="Guano 70"
                    /isolation_source="feces"
                    /host="Myotis yumanensis"
                    /db_xref="taxon:740971"
                    /geo_loc_name="USA: Texas"
    CDS             complement(<1..>201)
                    /codon_start=1
                    /product="DNA polymerase"
                    /protein_id="XDG10648"
                    /translation="DICGMYASALTHPFPAGRPLNPFDRALAVHRYEAKMKTCKTMDY
                    FDSALLPAIFTIDADPPDEHLLD"
ORIGIN      
       1 gtccagcaag tgttcgtccg gagggtcggc gtcgatggtg aagatggccg gcagaagagc
      61 ggagtcaaag tagtccatgg ttttgcaagt cttcatcttg gcttcgtacc tgtgcacggc
     121 gagcgctcgg tcgaacgggt tgaggggtcg ccccgcgggg aagggatgag tgagagccga
     181 agcgtacatg ccgcagatgt c
//
